# Use of in vivo confocal microscopy in suspected Acanthamoeba keratitis: a 12-year real-world data study at a Swedish regional referral center

**DOI:** 10.1186/s12348-024-00424-y

**Published:** 2024-09-10

**Authors:** Bogdana Toba, Neil Lagali

**Affiliations:** 1 Department of Biomedical and Clinical Sciences, Faculty of Medicine, Linköping University, Linköping, 581 83 Sweden; 2 Department of Ophthalmology, Sørlandet Hospital Arendal, Arendal, Norway

**Keywords:** Acanthamoeba, Keratitis, In vivo confocal microscopy, Cornea, Real-world data

## Abstract

**Purpose:**

To report real-world data (RWD) on the use of in vivo confocal microscopy (IVCM) in handling cases of suspected *Acanthamoeba* keratitis (AK) cases at a regional referral center during a 12-year period.

**Methods:**

Retrospective study of patients with suspected AK presenting at a regional referral center for IVCM in Sweden from 2010 to 2022. Demographics, symptoms, outcomes, and clinical management were analyzed, and IVCM images were interpreted.

**Results:**

Of 74 included patients with suspected AK, 18 (24%) were IVCM-positive, 33 (44%) were IVCM-negative, 15 had inconclusive IVCM results (20.2%), and 8 (11%) were referred for a second opinion based on IVCM, 4 of which were IVCM-positive (5.5%), yielding an overall IVCM-positive rate of 29.5%. Cultures were taken in 38 cases (51%) with only 2 cases (2.7%) culture-positive for AK. Of IVCM-negative cases, cultures were taken in 22 (67%) of cases and 100% of these were AK-negative. IVCM-positive cases had more clinic visits (median 30, *P* = 0.018) and longer follow-up time (median 890 days, *P* = 0.009) than IVCM-negative patients, while visual acuity improvement did not differ (*P* > 0.05). Of IVCM-positive cases, 10 (56%) underwent surgery despite prior anti-amoebic treatment, and 14 (78%) had 3 or more IVCM examinations during follow-up, with cysts (100%), dendritic cells (89%) and inflammatory infiltrate (67%) as the most prevalent features. Longitudinal IVCM indicated improvement in cysts, dendritic cells and subbasal nerves with treatment, while clinical resolution was not always consistent with complete absence of cysts.

**Conclusions:**

In a real-world setting, IVCM has a high reliability in classifying AK-negative cases, while IVCM detects AK-positive cases more frequently than the gold-standard culture method, leading to its preferential use over the culture method where time or resources are limited. Despite this, a subset of cases are IVCM-inconclusive, the clinical course of referred patients is long requiring many hospital visits, and visual acuity in most cases does not improve with medical treatment alone. Information sharing across centers and standardization of referral and diagnostic routines is needed to exploit the full potential of IVCM in AK patient management.

## Introduction

Acanthamoeba keratitis (AK) is a corneal infection caused by the *Acanthamoeba spp*. protozoa, a parasite that is ubiquitous in many water sources [1]. Several options exist for diagnosing AK including microbiological culture, real-time polymerase chain reaction (PCR) of a small corneal sample, and in vivo confocal microscopy (IVCM) examination of the cornea [2]. While generally considered a complimentary approach in the diagnosis of AK, IVCM has shown good diagnostic capabilities in multiple prior studies, reviewed in [3, 4]. Owing to its rapid diagnostic capability and availability, IVCM has been the main diagnostic approach used at our clinic on suspicion of AK, after performing thorough clinical examination and anamnesis.

The diagnostic and treatment process for patients with AK, however, is complex due to limitations in resources and specialist competence, resulting in patients often being treated in two to three clinics and receiving multiple therapies before an accurate diagnosis is established. A delay in correct diagnosis in many cases implies a greater risk for disease progression and infection of deeper layers in the cornea. One study has shown that with deeper corneal infection, there is a ten times greater probability of poorer visual acuity outcome [5]. Early AK detection is therefore critical, using a method having both high sensitivity and specificity. Tu et al. for instance, showed a sensitivity as high as 90.6% and specificity of 100% when comparing IVCM with corneal smears in 53 AK patients [6]. An additional advantage linked to the non-invasiveness of IVCM is the possibility of longitudinal evaluation of the therapeutic efficacy in the same patient [7–9].

Although IVCM has been shown to aid in diagnosing AK, most studies report carefully selected patient groups under controlled conditions, with the aim to determine the diagnostic accuracy of IVCM relative to another method, or to account for specific structures indicative of an AK infection [6, 8–14]. There is a lack of information, however, on how AK cases are handled in clinics using IVCM, apart from small case series. To the best of our knowledge only two reports describing treatment of AK in Sweden have been published, in 1996 and 2019 [15, 16]. Additionally, a larger study was conducted in the United States, however focusing only on a population of already diagnosed patients receiving treatment for AK [17]. We therefore aimed to conduct a real-world data (RWD) study to evaluate the use of IVCM in the clinical setting with suspected AK cases presenting at a single regional referral center in Sweden for IVCM. The study had two main objectives:

1. To retrospectively assess the characteristics and outcomes of patients with confirmed or suspected AK received at a regional referral center for IVCM examination.
2. To evaluate the clinical and morphological findings in cases positive for AK with IVCM, including longitudinal tissue changes visible in IVCM images after initiation of medical treatment.

## Methods

### Study design and ethics

This was a retrospective RWD study evaluating diagnosis and treatment of AK using IVCM at the Ophthalmology Department of the University Hospital in Linköping, Sweden. The study population was identified by retrospective review of completed IVCM examinations and medical journals of included patients. Ethical approval was obtained from the Swedish Ethical Review Authority prior to commencement of the study (Protocol No. 2021–05050).

### Patients and inclusion criteria

All included patients were either referred from their home clinic or initially visited the Ophthalmology Department in Linköping, Sweden (hereafter called the ‘referral center’) during a 12-year period (between September 2010 and January 2023). Inclusion criteria were the following: patients with either a distinct suspicion of AK after clinical and slit-lamp examination (including any combination of: clear infiltrate or ring-shaped infiltrate, staining epithelial defect, ocular pain, ocular redness, light sensitivity, reduced/fluctuating vision, excessive tearing, and/or history of contact lens wear or direct contact with water sources outside the home) or after non-responsiveness to treatments, and having an in vivo confocal microscopy (IVCM) examination performed and available for review at the referral center. IVCM examination and assessment of images was performed by a single experienced operator and grader, with 7–20 years of experience with IVCM examination and assessment of the cornea (including research in the field) during the study period. AK was formally diagnosed at the referral center based on clinical suspicion along with the presence of characteristic structures in IVCM images and/or positive culture result from the cornea, if a culture sample was taken. If bilateral involvement was suspected, both eyes were examined and included in the study. PCR for AK was not available as a diagnostic method at any of the referring clinics or at the referral center during the study period.

### Data collection and analysis

To facilitate demographic and outcome analyses, the following variables were recorded from the examined records: patient identifier (anonymized study number), sex, age, symptoms, logMAR visual acuity, risk factor(s), IVCM result, culture result, pharmacological treatment(s), previous treatment(s), other treatment received, clinical diagnosis, duration of care at the referral center (time from first to last visit), total number of visits, and final clinical outcome.

All cases where a culture sample was taken were quantified even if culture results indicated a different etiology, and pathogen(s) detected in the culture samples were recorded.

Patients were classified into one of the following four categories, based on their diagnostic status at the referral center:

1. IVCM-positive: AK diagnosis confirmed at the referral center by positive IVCM result.
2. IVCM-negative: Suspected AK diagnosis after slit lamp and anamnesis (as per inclusion criteria) that could not be confirmed by IVCM detection of characteristic positive features (see description below).
3. IVCM-inconclusive: unclear/inconclusive IVCM images where AK could not be ruled out at a given time point (at either first or subsequent IVCM examination).
4. IVCM 2^nd^ opinion: transferred to the referral center from another hospital for an IVCM examination to confirm or rule out a suspected AK diagnosis.

**Fig. 1:**
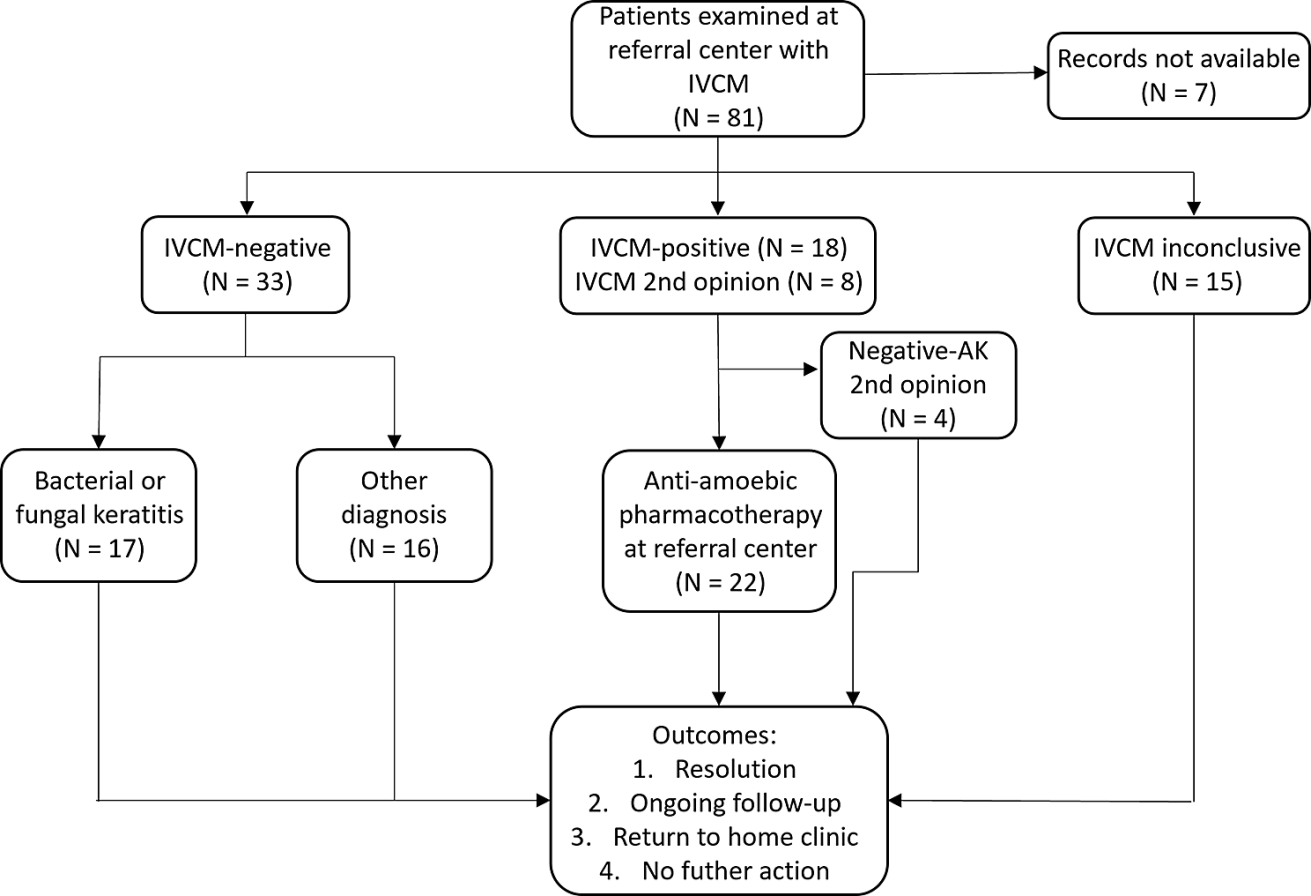
Flow chart describing the categorization and patient flow for patients referred to a single Swedish referral center for IVCM examination on suspicion of AK between September 2010 and January 2023

This categorization is illustrated in a flow diagram (Fig. 1). Regarding the IVCM-inconclusive category, examples of reasons for an unclear result included difficulty distinguishing AK structures from inflammatory cells or unclear or insufficient quality of IVCM images (often resulting from poor patient cooperation), precluding a reliable assessment.

### Outcome measures

During chart review, patient outcomes were noted according to the following classification:

- return to home clinic
- resolution
- no further action planned
- ongoing follow-up

Return to the home clinic could represent a single IVCM examination/patient visit to the referral center for confirmation purposes or may have involved multiple visits and treatments at the referral center, with subsequent monitoring at the patient’s home clinic. The requirement to be classified as ‘resolution’ or other regressing keratitis was an explicit record of the healing process as well as absence of recurrence. The category ‘no further action planned’ represented cases where resolution or complete healing was not explicitly stated in the patient chart, including cases where patients were given a medication regimen and instructed to return if problems arose but without further planned follow-up, or where an improvement was noted but without confirmation of full resolution (patient may have been sent to the home clinic upon improvement). Patients examined more recently and still under follow-up at the center at the time of analysis were classified as ‘ongoing follow up’.

### In vivo confocal microscopy (IVCM)

The scope of the image analysis was limited to patients with positive IVCM findings at the referral center. IVCM imaging of the cornea in all cases was performed by a single operator for the 12-year study period (author NL) using the HRT3 with Rostock Corneal Module confocal microscope (HRT3-RCM, Heidelberg Engineering, Heidelberg, Germany), with the same operator performing all examinations. The examination generated a series of images, each representing an area of 400 × 400 μm, with a typical examination consisting of approximately 1000 images. From these, the experienced IVCM assessor selected a total of 10–15 non-duplicate images from each eye at the relevant depth (epithelium, subbasal plexus, stroma) that were representative of morphological features for the examination. For image analysis, six morphological structures were identified as indicative of AK: cysts, suspected trophozoites [18], inflammatory infiltrate [11, 19], ulcerations (area without corneal epithelium corresponding to fluorescein staining region), presence of dendritic cells, and reduced or absent subbasal nerves. Cysts could exhibit diverse forms, including a pre-cystic appearance, a double-walled cyst structure, ‘target sign’ appearance, cluster formations of circular brightly reflective structures, chains, and other configurations which could indicate other active or inactive cysts [20]. In this study, trophozoites were classified as “suspected” due to the inherent difficulty in distinguishing these from other hyperreflective structures. The image evaluation process relied on experience, prior research and discussions with experts, and subjective judgement. Confounding factors often observed in AK patients were also considered, such as inflammatory cells/infiltrate with cyst-like appearance and various artefacts.

Images were semi-quantitatively assessed. Ulceration, trophozoites, and inflammatory infiltrate were assigned a score of 0 indicating absence or 1 indicating presence. Other features such as cysts, target sign, dendritic cells and nerves were assessed semi-quantitatively: 0 - absence, I - low abundance, II - moderate abundance, and III - high abundance. Patients with cysts were graded as I if 1 or 2 cysts were identified per analyzed IVCM image; II − 3–10 cysts per image; and III - >10 cysts per image. Nerves were graded as I if 1 or 2 distinct subbasal nerves were identified per analyzed IVCM image; II − 2, 3 or 4 nerves present in the image; and III - ≥ 5 nerves per image. Dendritic cells were graded as I if ≤ 5 were present in the image; II − 6–20 present; and III - > 20 present. In addition, the morphology, distribution and density of the above features were compared before and after anti-amoebic therapy, focusing on the corneal region with clearly defined epithelial defect area where suspected features were located. A single evaluator with prior experience assessing IVCM images performed image grading with additional training, guidance and oversight from the experienced grader who performed all IVCM patient examinations at the referral center. All grading was performed without knowledge of pre/post therapy status and was checked for accuracy by the experienced grader. Any unclear structures that were difficult to interpret were subjected to repeated assessments and discussions to ensure accurate interpretation and agreement. Data was recorded directly into an Excel spreadsheet.

### Statistical analysis

Statistical analysis was performed with SPSS software (IBM SPSS Statistics V24, IBM Corp, Armonk, New York, USA). Descriptive statistics were used to summarize patient demographic data. Nominal variables such as patient outcome, sex and other treatments were coded numerically to simplify further analysis. Normally distributed data are presented as mean and standard deviation (SD), and non-normally distributed data as median and range. Age, length of follow-up, and number of visits were non-normally distributed and further analyzed across groups with the independent-samples Kruskal-Wallis test. Based on the distribution of initial and final visual acuities (VA), values were compared for each patient group using the Paired t-Test or Wilcoxon signed rank test. VA changes across patient groups were further analyzed with the nonparametric Kruskal-Wallis test. Symptoms at the first visit and differences in outcomes across patient groups were analyzed using the Chi-Square Test. For IVCM image analyses, the Pearson Chi-Square, Wilcoxon and independent samples t-tests were used to assess statistical significance. In all cases, a two-tailed P value of < 0.05 was considered statistically significant.

## Results

### Study population

Examination of microscopy records yielded 81 consecutive patients undergoing IVCM examination for suspected AK at the referral center during the 12-year study period. Substantially more patients with suspected or confirmed diagnosis of AK were present in the medical records during the study period, but these were not referred for IVCM. Of the 81 identified patients who underwent IVCM, 7 (8.6%) lacked corresponding medical records and were thus excluded (Fig. 1). Six of these excluded cases came to the referral center for IVCM exam only, with all medical management being performed by the referring clinic. The remaining excluded case was an inclusion error as the patient changed name between visits and was mistakenly assigned two anonymization codes.

For the 74 included cases, the median age was 60.5 years (range: 33–70 years) with 40 (54%) males and 34 (46%) females. Most patients had a unilateral presentation of keratitis, with only 2 bilateral cases (2.7%), where both eyes were included in the IVCM examination, yielding a total of 76 eyes included in the study. Demographics for the study groups are given in Table 1. The largest group consisted of those with IVCM-negative findings for AK (44%), followed by IVCM-positive (24%), unclear/inconclusive IVCM findings (20%) and referrals for second opinion on diagnosis using only IVCM (11%), of which half were IVCM-positive for AK, yielding a total IVCM-positive cases of 22 (29.5%) and second opinion negative cases to 4 (5.5%).

### Length of care and clinic visits

The days under care and number of visits at the referral center varied considerably across the subgroups. Excluding patients with a single visit for IVCM without further follow-up at the referral center (14 cases, 19%), the median values for days of care and number of visits were determined (Table 1). Length of care ranged from 3 to 3092 days, while the number of clinic visits for a patient ranged from 2 to 73. Both parameters were highly variable across all four categories.

Patients referred to the center for a second opinion (and thereafter treated at the referral center) had the longest median follow-up time (2252 days) while the IVCM-negative cases had the shortest follow-up time (99 days). The IVCM-positive group had the most visits (median of 30 visits). A total of 9 patients (12%) required inpatient care for various reasons; either to carry out a surgical procedure or for assistance with eye drop therapy (typically in the elderly) as this required strict adherence to the treatment schedule. No significant difference in demographic characteristics across patient categories was noted for the entire group. Comparing the IVCM-positive with the IVCM-negative cases, however, it was found that significantly fewer days of care and number of visits were required for IVCM-negative cases relative to IVCM-positive cases (Table 1).

**Table 1:** Demographic characteristics of the study participants

Patient category (number of subjects)	Age Median (SD)	Days of care Median (SD)	Number of visits Median (SD)
All (74)	60.5 (13.2)	233 (730)	11.0 (13.7)
IVCM-negative (33)	33.8 (15.2)	99 (200)	18.0 (11.3)
IVCM-positive (18)	53.3 (10.5)	890 (937)	30.0 (22.5)
IVCM-inconclusive (15)	50.1 (17.2)	1252 (291)	11.0 (8.2)
IVCM 2nd opinion (8)	80.4 (28.2)	2252 (1087)	22.0 (9.9)
ANOVA Kruskal-Wallis P-value (all groups)	0.44	0.05	0.56
Mann-Whitney P-value (IVCM-positive vs. negative)	0.32	**0.009**	**0.018**

SD = standard deviation

### Symptoms and predisposing factors

At the first visit to the referral center, the most prevalent symptoms were pain (32 cases, 43.2%), irritation/itching sensation (28 cases, 37.8%), visual impairment (24 cases, 32.4%), light sensitivity/photophobia (15 cases, 20.3%), redness (12 cases, 16.2%) and lacrimation/excessive tearing (9 cases, 12.2%). Other less frequent symptoms included eyelid swelling, pus, burning sensation, and dryness. Symptoms did not differ in frequency across the four categories (Pearson Chi-Square, *P* = 0.80). The most predominant predisposing factor for referral was contact lens wear (42 patients; 57%). Other predisposing factors included exposure of the eyes to various water sources as lakes and pools (5 patients; 6.8%), direct eye trauma (4 patients; 5.4%), exposure to bath/shower with contact lens in place (4 patients; 5.4%), and cataract surgery (4 patients; 5.4%). Less frequent predisposing factors were corneal transplantation, keratoconus/ectasia and DSAEK surgery.

### Culture results

Although all included patients underwent IVCM examination at the referral center, 38 cultures were taken (51% of the population), of which only 2 of 38 (5.3%) were positive for *Acanthamoeba* and 17 of 38 (45%) indicated a different etiology, while the remaining 19 samples (50%) were culture negative. Of the 22 cases where IVCM was positive for AK, treatment was initiated immediately following the IVCM-positive result, but culture samples were taken in only 9/22 cases (40.9%). Of these 9 cases, only two were culture-positive for AK. Of the 33 IVCM-negative cases handled at the referral center, culture samples were taken in 22/33 (66.7%), and all 22 (100%) of these were culture negative for AK, with 11/22 (50%) positive for other pathogens.

### Treatment

18 of the 74 included patients (24%) were diagnosed with AK while 22 (30%) received AK treatment at the referral center consisting of a combination treatment with Brolene and Chlorhexidine or PHMB alone. The 4 patients treated at the referral center who were not formally diagnosed with AK received treatment based on clinical evidence. Several of the included patients underwent additional surgical treatments including collagen crosslinking (7 patients; 9.5%), amniotic membrane transplantation (6; 8.1%) and corneal transplantation (6; 8.1%). 4 of 7 crosslinking cases (57%), 3 of 6 amniotic membrane transplantations (50%) and 3 of 6 corneal transplantation (50%) were performed in the IVCM-positive group, indicating that in about half of the cases pharmacological treatment alone was insufficient to eradicate the parasite and/or to recover adequate vision. In total, 2 eviscerations were performed, and these were also within the IVCM-positive group, indicating a rate of 2 of 74 (2.7%) of all suspected AK cases subjected to IVCM examination during a 12-year period.

### Visual acuity

A subset of patients lacked available VA values in their medical records. Additionally, not all measurements were directly related to the (suspected) AK condition. A total of 48 of 74 patients (65%) had initial and final VA in their records. Change in VA was calculated as final minus initial VA taken from first and last clinic visits, where a positive logMAR difference value indicated deterioration of vision and negative logMAR an improvement. Nonparametric Kruskal-Wallis analysis across categories revealed no significant difference in initial, final or change in VA across the four categories or between IVCM-positive and IVCM-negative patients (*P* > 0.05 for all, Table 2).

**Table 2:** Median values of initial, final and change in visual acuity (VA) for each patient category

Category	Initial VA Median (range)	Final VA Median (range)	VA change (final-initial) Median (range)	Wilcoxon *P*-value^1^	Number of subjects
Entire population	1.3 (0,3)	0.7 (0,3)	0.0 (-2,2)	0.30	48
IVCM-negative	1.0 (0,2.3)	0.5 (0,2.7)	0.0 (-1.9,1)	0.22	21
IVCM-positive	1.9 (0,3)	1.5(0,3)	0.0 (-1.8,2)	0.80	15
IVCM-inconclusive	2.0 (0,2.7)	1.0 (0,2.7)	0.0 (-2,1)	0.54	7
IVCM 2nd opinion	1.9 (0.3,2)	1.4 (0.1,3)	0.0 (-0.9,1)	1.00	5
Kruskal-Wallis Test across categories	0.71	0.06	0.80		
Wilcoxon P-value (AK-positive vs. AK-negative)	0.29	*0.12*	0.40		

^1^Initial VA versus final VA

### Outcomes

In total 71 of 74 patients (96%) had data available for outcome analysis (Table 3). A total of 50 of 71 cases (70.4%) did not have a recorded final outcome at the department because of returning to their home clinic or being classified as ‘no further action planned’ due to changing clinic, moving to another region, or simply being instructed to contact the department again if necessary. 37 patients (52%) referred to the department for IVCM returned to their home institution where AK or another keratitis was subsequently managed. Of the 34 patients remaining under care at the department, a total of 20 cases of keratitis resolution were recorded (59%), consisting of AK, bacterial and fungal cases. No significant differences in outcome distributions were noted across the four patient categories (*P* = 0.19, Pearson Chi-Square test).

**Table 3:** Clinical outcomes across patient categories

Category	Number of patients					
	Entire population		IVCM-negative	IVCM-positive	IVCM inconclusive	IVCM second opinion
Return to home clinic	37		17	8	7	5
Resolution	20		11	5	4	0
No further action	10		3	3	1	3
Ongoing follow up	4		0	2	2	0
Chi-Square P-value		0.19				

### In vivo confocal microscopy

Of the 18 patients in the IVCM AK-positive category, 8 (44%) were referred for further follow-up at the home clinic, without final outcome data available. Among the 10 patients remaining under departmental care, 8 (80%) had the AK infection resolved or required no additional follow-up at the department, while 2 (20%) were still being monitored at the time of analysis. All 18 patients received pharmacological AK treatment, with 10 (56%) also undergoing complementary surgical interventions. Most patients (78%, 14/18) underwent 3 or more IVCM examinations during their follow-up at the department. IVCM structures found at the first visit, last visit or at least once during the entire follow-up period are presented in Table 4. The most prevalent features observed at least once in the images of IVCM-positive patients were cysts (100%), dendritic cells (89%) and inflammatory infiltrate (67%). Notably, 6 patients (33%) at the final visit still had cysts/target signs present upon IVCM examination, with some of these patients being sent back to their home clinic and not remaining under care at the department.

**Table 4:** Frequency of IVCM morphologic features detected in 18 IVCM-positive AK patients

Structure	Frequency of detection – number of patients (%)		
	First visit	At least one visit	Final visit
Cysts	17 (94)	18 (100)	6 (33)
Dendritic cells	11 (61)	16 (89)	5 (28)
Inflammatory infiltrate	9 (50)	12 (67)	5 (28)
Subbasal nerves	5 (28)	10 (56)	7 (39)
Suspected trophozoites	4 (22)	8 (45)	3 (17)
Ulcer/lesion	4 (22)	7 (39)	2 (11)

A group of 16 diagnosed AK patients treated at the referral center, either until resolution of their infection, no further follow up, or return to their home clinic was examined more closely to identify potential patterns in their IVCM data during their time at the referral center (Table 5). Only one patient under care at the referral center underwent a single IVCM examination that revealed clear grade III cysts, yet no subsequent IVCM examination was conducted, for unknown reasons. Determination of resolution in this case was instead reliant solely on the clinical presentation. In the 7 patients under care at the referral center, improvement in IVCM features was noted from the first to the final IVCM evaluation. Not all patients, however, had complete absence of cyst structures at the final examination. For example, one patient had grade II cysts at the final examination but the subbasal nerves recovered (grade 0 to III) and dendritic cells declined (grade III to 0). Despite the presence of residual cysts, the outcome was considered as a resolution, considering the other IVCM changes and clinical assessment. In these cases, resolution of AK clinically was not consistent with complete absence of cyst structures on IVCM examination.

**Table 5:** Longitudinal IVCM findings in 16 AK-positive patients indicating most severe grade and grade at final IVCM examination1

Patient no.	Ulcer^2^	Cysts	Dendritic cells	Inflammatory infiltrate^2^	Subbasal nerves	Suspected Trophozoites^2^	Total IVCM^3^
**Patients treated at the referral center until resolution**							
1	0	III to 0	II to 0	0	II to III	0	3
2	I to 0	III to 0	III to II	I to 0	0 to I	0	6
3	I to 0	I to 0	III to 0	I to 0	I to III	0	4
4	0	III to 0	I to 0	I to 0	III to 0	0	7
5	I	III	0	0	0	0	1
6	I to 0	III to II	III to 0	I to 0	0 to III	0	5
7	0	I to III	III to I	I to 0	0 to III	0	4
**Patients treated then referred back to their home clinic**							
8	0	I to 0	I to 0	0	0 to I	I to 0	4
9	0	III	0 to III	I	0	I to 0	5
10	0	I to 0	I to 0	0	0	I to 0	4
11	0	III	I to III	0 to I	0	0	2
12	0	II to I	0	I	0	0 to I	3
13	0	II to 0	0	I to 0	II to 0	I to 0	5
14	I to 0	II to 0	II to 0	I	0	0	4
15	0	III	I to 0	0	0	I to 0	4
16	I to 0	II to III	II to III	0	III to 0	0 to I	2

^1^a single grade indicates the grade was unchanged throughout the course of examinations

^2^ulcers, inflammatory infiltrate and trophozoites were graded on a binary scale with ‘I’ indicating presence and ‘0’ indicating absence

^3^total number of IVCM examinations performed at the department

**Fig. 2:**
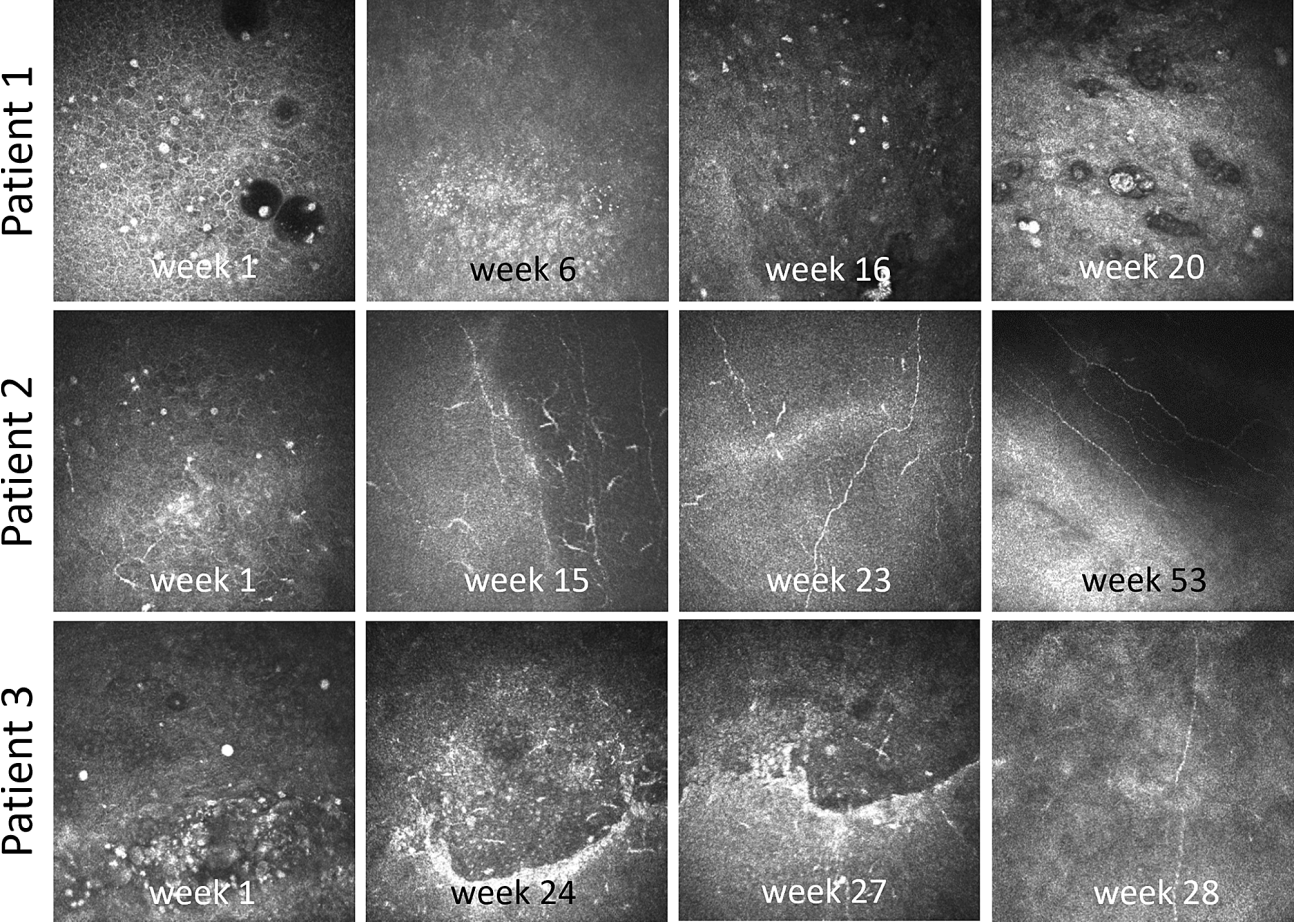
Longitudinal in vivo laser confocal microscopy (IVCM) images in three patients treated at the referral center. These images exemplify changes observed during treatment, from initial IVCM examination to resolution of the keratitis, with the time of examination in weeks after first presentation indicated. **Patient 1**: demonstration of change in cyst density, size and morphology during the treatment period. **Patient 2**: illustration of change in the degree of subbasal nerves and dendritic cells during treatment. **Patient 3**: change in status of the epithelial lesion/ulcer during the treatment period. Note: images were selected from four representative visits and do not represent all IVCM examinations for these patients

Patients with complete AK resolution at the referral center exhibited a reduction in cysts and dendritic cells and increase in subbasal nerves from the first to last visits. One case of resolution, however, had increased cyst grade at the final IVCM (I to III). Nonetheless, for this patient there was an improvement in the nerve score from 0 to III and in the dendritic cell score from III to I. It should be emphasized that patients were deemed to have achieved resolution after further clinical follow-up without IVCM examinations, thus without complete knowledge of cyst presence. A reciprocal relationship between dendritic cells and nerves was also noted. In Fig. 2, representative cases are shown to illustrate changes observed during repeated IVCM examinations.

The subgroup of 9 patients diagnosed with AK after IVCM examination but later sent to their home clinic for further management were also examined. Table 5 indicates that two of these patients left the department with residual suspected trophozoites and 5 patients left with residual cysts. No difference in cyst presence was observed, however, across the subgroupings shown in Table 5 (*P* = 0.69). Although dendritic cells also did not differ across the subgroups (*P* = 0.38), the inflammatory infiltrate in the cornea had not resolved in about half of the patients sent back to their home clinic. Furthermore, only one patient in this subgroup had visible nerves during the final IVCM examination at the department.

## Discussion

In contrast to prior studies limited to evaluating IVCM for detection and diagnosis of AK often in relation to other diagnostic methods, the present investigation was a RWD study aimed at highlighting how IVCM examinations have been incorporated into clinical practice at a regional referral center for IVCM. While the retrospective study design and focus on a single center revealed incompleteness of information prior to arrival or after discharge from the referral center, a major strength of the study is that it highlights the use of IVCM in handling suspected AK cases in a real-world setting outside the controlled conditions of a clinical study, where special measures are made to obtain as complete datasets as possible. Most prior studies also report a short follow-up duration for patients, without clear timelines for AK resolution. In Sweden in particular, the only published series involving AK (but not IVCM) describes 8 patients treated over 30 years ago [15]. Given the escalating usage of contact lenses and the increasing incidence of AK cases in recent years, assessing the current real-world diagnosis and treatment of patients with suspected AK in centers with IVCM is essential. This study highlights key areas for improvement in the integration of IVCM into a clinical setting where multiple hospitals may refer patients to a single regional referral center with expertise in IVCM.

Overall, our referral center examined 81 suspected AK patients in a 12-year period with IVCM. Not all cases were well-documented, likely because patients were sometimes referred for the sole purpose of performing an IVCM examination and reporting results to the home clinic. For referrals from another hospital requesting a second opinion, half were IVCM-positive and treatment was initiated at our referral center, with the other half being IVCM-negative and directly returning to the care of the home clinic. These cases could be followed up with the referring clinic in the future, to better understand the clinical outcomes related to the IVCM-based diagnoses.

A large group of referred patients had IVCM-negative and inconclusive findings, and of these cases many were bacterial keratitis. For those cases not treated at our center, future follow-up to determine the final diagnosis would aid in evaluating the features observed by IVCM. Regardless of patient category in this study, however, most patients received some form of care at our center prior to discharge or return to the home clinic. The median time of care and number of visits to our center were highly variable but were generally extensive. Typically, the management of patients with suspected AK lasted years, not months, with the entire group of patients having a median of 11 clinic visits during the study period, which does not include prior or subsequent visits at the home clinic. The group with the longest median management time (about 6 years) was the referred to our center for a second opinion and subsequently managed at our center. These were cases of delayed diagnosis or misdiagnosis, and/or treatment-resistant cases with repeated sequelae and co-morbidities, where patients often underwent multiple surgical interventions with long follow-ups. This illustrates the complexity of managing AK cases in the real-world setting, and the significant clinic resources allocated to this patient group. Nevertheless, we found a significantly shorter time of care and fewer clinic visits for IVCM-negative cases relative to IVCM-positive cases. Moreover, 100% of IVCM-negative cases who had culture samples taken were also culture-negative, representing a 100% negative predictive value for IVCM at the referral center. This highlights that IVCM examination can successfully predict those cases which are likely not AK and will thus have a shorter clinical course and require fewer resources.

While 57% of the population were documented contact lens wearers, the proportion may have been higher as the etiology was not systematically recorded in the patient charts. Ideally, all patients with suspected AK would also, in addition to IVCM, have samples taken for culture and/or PCR. This reflects the different practices at different centers and in different countries, and the real-world situation where not all diagnostic modalities may be available, or where time may be of the essence, particularly with a patient receiving a positive diagnosis then returning to the referring clinic. Even where multiple diagnostics are available, the treating physician may choose not to employ all of them, particularly where IVCM indicates a clear positive result or where resources are limited. At the referring centers and at the IVCM referral center during the time frame of this study, PCR was not implemented as a diagnostic technique (but has later been adopted at larger academic centers in Sweden), and cultures were taken in only about half of the cases at the referral center, in contrast to controlled studies evaluating the diagnostic accuracy of IVCM, which have typically shown a good diagnostic accuracy [4], although this can be dependent on the level of experience of the IVCM operator [21]. In the real-world setting at our center, a positive IVCM finding resulted in the patient immediately receiving anti-amoebic therapy without culture samples typically being taken. Also, culture was performed mostly in cases not responding to first-line pharmacologic treatment. In these cases, the clinical picture, patient history/predisposing factors and IVCM findings were sufficient for initiation of treatment. The typically long delay in receiving culture results and typically low culture positivity for AK may have also played a role in the decision not to take a culture sample. On the other hand, a negative IVCM finding often resulted in either a transfer back to the referring center or in some cases a culture sample being taken. Reliance on culture results was not high at our center, and this may partly be due to the finding that only 2 cases were culture-positive for AK whereas 22 cases were IVCM-positive for AK. Moreover, half of the culture samples were negative for pathogens of any kind. Drawbacks of the culture method have previously been shown in other studies, possibly due to the culture sample not containing any cysts [20, 22]. It may also be possible that the culturing method used may not be adequate or that previous applied therapies may impede the ability to culture the parasite. Time constraints also play a role as cultures typically require a time frame of 2 days to 2 weeks, and the proportion of positive results varies widely, ranging from 0 to 68%, which is also a major disadvantage [23, 24].

Evaluation of images from IVCM-positive cases always revealed cysts on at least one visit (100%), and additionally dendritic cells (88.9%) and a clear inflammatory infiltrate (66.6%). It should be kept in mind, however, that cyst identification is done by humans, and although machine learning tools are being developed [25], a risk for false positives remains [20]. The analysis indicated that patients returning to their home institutions exhibited more severe inflammation (*P* = 0.04) and worse corneal nerve status (*P* = 0.004) at their final IVCM examination at our referral center compared to those remaining under care at the referral center until resolution. This likely reflects the active infection and inflammation still present upon discharge, which is expected to improve with continued patient management at the home clinic. It is important to note that the presence of cysts in repeated IVCM examinations does not necessarily indicate therapy resistance or lack of improvement, as the activity of these cysts could not be determined. Previous studies have shown that infection resolution does not always eliminate cysts [8, 26, 27]. Therefore, an integrated assessment of all clinical characteristics is necessary to determine the clinical significance of findings of residual corneal cysts.

The relatively long time of care in this study was often the result of a long patient history prior to referral to our center, often characterized by multiple prior diagnoses, and for this reason the study may be biased towards reporting the most difficult and complex cases. These cases, however, are those typically sent to our regional referral center, which is part of an academic hospital. In Sweden (with a population of 10.5 million), only 4 centers are currently using IVCM. Unfortunately, delayed initiation of appropriate treatment can have a significant impact on prognosis, as it has been reported that starting treatment after one month of symptom onset leads to greater morbidity and poorer visual outcomes [28]. A study involving 105 AK patients showed that early diagnosis resulted in a final visual acuity of 6/12 or greater in over 90% of cases, while delayed diagnosis resulted in this level of vision in only 65% of cases [29]. In addition, more advanced deeper infections are ten times more likely to be associated with poorer VA [5] and the need for subsequent surgical interventions [30]. Therefore, a possible solution could be a centralized approach to manage suspected and diagnosed AK cases at a single center experienced in the use of IVCM. This would require better education and outreach to the referring centers that do not use IVCM.

Notably, we did not find differences in VA from the first to the final patient visit at our center, regardless of the patient category. This may be due to the high proportion of severe keratitis cases referred to our center, where vision improvement is often secondary to the eradication of the causative pathogen. VA measurements, however, also constitute a limitation in our study. Aside from lack of complete visual acuity data in all patients, acuity values in the patient records were measured by different physicians often with varying levels of accuracy. Ocular comorbidities impacting vision further complicate the representation of VA as an outcome measure of AK treatment.

In this study we identified a subgroup of 14 patients (19%) who visited the clinic for a single IVCM examination with the sole purpose to confirm or rule out AK diagnosis. It is important to note that relying on a single examination may not capture pathognomonic features such as cysts, particularly in the early stages of the disease when a dense inflammatory infiltrate may be present. Moreover, a previous study highlighted the advantages of follow-up IVCM examinations [8], where the density of cysts during the early stages of the disease can be minimal, with the highest density occurring approximately two weeks after initiating AK treatment, followed by a clear reduction after about 2 months of treatment. This peak in cyst numbers is attributed to the two-cycle life form of *Acanthamoeba*, where trophozoites convert into cysts in response to the treatment-induced unfavorable microenvironment. Therefore, only a short-term assessment of patients could lead to erroneous conclusions and suboptimal management including unnecessary surgery [8]. Longer term evaluation with IVCM may serve as a reliable indicator of the patient’s responsiveness to AK treatment, based not only on cysts but on other signs of healing such as regeneration of subbasal nerve fibers, resolution of the inflammatory infiltrate, healing of the ulcer (return of epithelial cell mosaic), and a lower grade of dendritic cells.

The results of this study also suggest a clear need for better documentation since information can be transmitted verbally between treating centers and may not be adequately documented in the medical record system (for example, with a formal referral letter) when patients are referred. Moreover, the medical record system in Sweden is region-based; to obtain full patient history and outcomes for patients outside the region of the referral center would require specific research ethics, collaboration and data sharing agreements across multiple regions and hospitals. Additionally, relying on a single IVCM examination may prove insufficient in ruling out an AK diagnosis, instead multiple examinations are desirable. Follow-up of patients with repeated IVCM examinations, while highly variable in this real-world setting, could in the future be better prioritized and standardized.

Given the results and limitations of this study, we recommend the following measures to be taken by referral centers for IVCM in Sweden receiving suspected AK cases:

1. A comprehensive system for monitoring and recording clinical information from patients with a positive IVCM diagnosis for AK across referring and referral centers is needed; if this is hindered by different region-based systems and regulations on data sharing, an alternative is to develop a national registry for AK-positive cases. In this way, all information regarding the AK diagnosis and treatment for patients would be available in a single place, regardless of which clinics or regions the patient visits.
2. Not all cases of suspected AK are examined with IVCM. It is recommended that upon suspicion of AK, regardless of whether a culture sample or swab is taken, the patient is referred for IVCM examination. Given the many clinic visits and long time to resolution for AK, the benefits would likely outweigh the extra cost for referral, given that AK is a relatively rare but potentially devastating condition. This recommendation may involve targeted outreach and communication to clinics where IVCM is not available.
3. Strict protocols should be implemented for documentation of contact lens wear and other risk factors in patient records in cases of suspected and confirmed AK.
4. Upon detecting positive evidence of AK by IVCM, referral centers should communicate the preferred treatment regime to the treating physician, which is of particular importance when patients return to the home clinic for subsequent management. Consistency of first-line treatment regimens could improve outcomes.
5. Referral centers should take culture and PCR samples in addition to performing IVCM, if the referring center had not initiated these complementary diagnostics.
6. In cases of positive IVCM diagnosis, the referral center should recommend further follow-up visits for IVCM even if the patient returns to the home clinic for medical management.

In summary, to the best of our knowledge, this is a unique real-world study evaluating all suspected AK cases referred for IVCM during a 12-year period. IVCM had a 100% negative predictive value that also mirrored a shorter clinical course to keratitis resolution and coincided with positive culture detection of other pathogens in these AK-negative cases. Importantly, positive IVCM detection of AK was much more frequent than culture positivity for AK. Direct instatement of anti-amoebic medical therapy upon positive IVCM findings led to AK resolution in many cases, although the time to resolution was long, given the advanced nature of AK and long clinical history of patients seen at the regional referral center. Additionally, a group of suspected AK cases were inconclusive by IVCM, which is an important group and highlights the practical limitations of IVCM and the continued need for multiple clinical diagnostics and follow-ups in cases of suspected AK. Improved communication and information sharing between referring clinics and the referral center could yield benefits for patients by potentially reducing the time to diagnosis and aiding in ongoing patient management. Standardization of documentation and diagnostic routines in the management of AK, as well as spreading of information across regions about IVCM as an available diagnostic method could also yield improved care for patients and better exploitation of the utility of IVCM as a complementary diagnostic tool in a real-world context.

## Data Availability

The datasets generated and/or analysed during the current study are not publicly available as they contain sensitive personal and clinic information pertaining to health status and clinical management of cases.

## Abbreviations

ANOVA: Analysis of variance
AK: Acanthamoeba keratitis
HRT3-RCM: Heidelberg Retinal Tomograph 3 – Rostock Cornea Module
IVCM: In vivo confocal microscopy
logMAR: logarithm of the minimum angle of resolution
PCR: Polymerase chain reaction
PHMB: Polyhexamethylene biguanide
RWD: Real-world data
SD: Standard deviation
VA: Visual acuity
